# FN1, SPARC, and SERPINE1 are highly expressed and significantly related to a poor prognosis of gastric adenocarcinoma revealed by microarray and bioinformatics

**DOI:** 10.1038/s41598-019-43924-x

**Published:** 2019-05-24

**Authors:** Li Li, Zuan Zhu, Yanchao Zhao, Qi Zhang, Xiaoting Wu, Bei Miao, Jiang Cao, Sujuan Fei

**Affiliations:** 1 Department of Gastroenterology, the Affiliated Hospital of Xuzhou Medical University, Xuzhou, Jiangsu 221000 China; 2 Department of Hematology, the Affiliated Hospital of Xuzhou Medical University, Xuzhou, Jiangsu 221000 China

**Keywords:** Gastric cancer, Prognostic markers, Oncogenes

## Abstract

Gastric adenocarcinoma (GAC), also known as stomach adenocarcinoma (STAD), is one of the most lethal malignancies in the world. It is vital to classify and detect the hub genes and key pathways participated in the initiation and progression of GAC. In this study, we collected and sequenced 15 pairs of GAC tumor tissues and the adjacent normal tissues. Differentially expressed genes (DEGs) were analyzed and Kyoto Encyclopedia of Genes and Genomes (KEGG) pathway and Gene Ontology (GO) analysis were used to annotate the unique biological significance and important pathways of enriched DEGs. Moreover, we constructed the protein-protein interaction (PPI) network by Cytoscape and conducted KEGG enrichment analysis of the prime module. We further applied the TCGA database to start the survival analysis of these hub genes by Kaplan-Meier estimates. Finally, we obtained total 233 DEGs consisted of 64 up-regulated genes and 169 down-regulated genes. GO enrichment analysis found that DEGs most significantly enriched in single organism process, extracellular region, and extracellular region part. KEGG pathway enrichment analysis suggested that DEGs most significantly enriched in Protein digestion and absorption, Gastric acid secretion, and ECM-receptor interaction. Furthermore, the PPI network showed that the top 10 hub genes in GAC were IL8, COL1A1, MMP9, SST, COL1A2, TIMP1, FN1, SPARC, ALDH1A1, and SERPINE1 respectively. The prime gene interaction module in PPI network was enriched in protein digestion and absorption, ECM receptor interaction, the PI3K-Akt signaling pathway, and pathway in cancer. Survival analysis based on the TCGA database found that the expression of the FN1, SERPINE1, and SPARC significantly predicted poor prognosis of GAC. Collectively, we identified several hub genes and key pathways associated with GAC initiation and progression by analyzing the microarray data on DEGs, which provided a detailed molecular mechanism underlying GAC occurrence and progression.

## Introduction

Gastric cancers are the fifth commonest cancer after lung, breast, colorectal and prostate cancers^1^. It imposes a considerable health burden worldwide. Gastric adenocarcinoma (GAC), also known as stomach adenocarcinoma (STAD) is the commonest histological type (~90–95%)^2^. In 2015, GAC was expected to be diagnosed nearly 777,000 new cases and led to deaths of 350,000 people worldwide^3^. Although advances have been made for the diagnostic and therapeutic techniques for decades, the mortality rate of GAC is still high and the global 5-year survival rates remain unsatisfactory^4^.

It is well known that cancer is usually characterized by abnormally cell cycle activity, which generally results from either mutation in the up- or down-stream signaling pathways or genetic lesions in protein-encoding genes involved in cell cycle. The highly organized and regulated mammalian cell cycle ensures normal and accurate gene duplication, cell division and cell apoptosis^5^.

Microarray could be used to probe the key biomarkers and provide a better understanding of the molecular mechanisms involved in GAC. Until now, clinically applicable biomarkers are still lacking. Therefore, exploring novel and effective molecular biomarkers to elucidate effective therapeutic targets for GAC patients is still imperative. In this study, we focused on the different expression pattern between the GAC tumor tissues and matched normal tissues. To discover the hub genes and key pathways associated with the initiation and progression of GAC, we applied differential gene expression analysis and functional enrichment analysis. In conclusion, we identified a set of hub genes that participated in several cancer-relevant pathways and their abnormal expression are correlated with the clinical prognosis of GAC people by overall survival analysis.

## Methods and Materials

### Patients and samples

Tumor and matched normal tissues samples were obtained from the GAC patients at the Affiliated Hospital of Xuzhou Medical University in 2014. These tissues were stored in RNAlater (Ambion, Life Technologies, ThermoFisher Scientific, Waltham, MA, USA) at 4 °C until full penetration of RNAlater into the tissues and transferred to −80 °C for storage. The selection criteria were as follows: (1) the subject presented was diagnosed as GAC and no history of other tumors; (2) Complete demographic and clinical data including age, gender, clinical manifestations, tumor size, the extent of resection, and date of relapse and/or death have been collected. In order to get the formal permission of surgical procedures and the intelligent use of the resected tissues, the legal surrogates of those participants provided their Written informed consent. The National Regulations on the Use of Clinical Samples in China is as a guideline for human tissue acquisition and legitimate use. This study was approved by the Medical Ethics Committee of the Affiliated Hospital of Xuzhou Medical University. The demographic and clinical features of the patient were summarized in Table 1.

**Table 1:** The demographic and clinical features of the patient.

**Gender**	
Male	20 (~67%)
Female	10 (~33%)
**Age**	
Median	43
Range	23–66
Race	EthnicHan
**Stage**	
I	5 (~17%)
II	8 (~27%)
III	11 (~37%)
IV	6 (~20%)

### Profiling of gene expression

Total RNA was extracted from frozen tissues separately using EZNA ® HP Tissue RNA Kit (Omega Bio-Tek Inc., Norcross, GA, USA) according to the manufacturer’s recommended procedure. Bioanalyzer 2100 (Agilent Technologies, Palo Alto, Calif.) was used to assess the quality and quantity of these total RNAs. Affymetrix microarray was used for mRNA profiling, which was performed by GeneChem (Shanghai Genechem Co., Ltd.). Briefly, after rRNA removal, biotinylated aRNA (cRNA) was prepared according to the manufacturer’s protocol (3′ IVT Express Kit, Affymetrix 901228). PrimeView Human Gene Expression Array (cat. no. 901838; Affymetrix; Thermo Fisher Scientific, Inc.) were hybridized and scanned according to standard Affymetrix protocols. All samples were processed in technical duplicate. GeneChip Scanner 3000 (Affymetrix) was used to scan the completed arrays. Images were extracted with Affymetrix GeneChip Command Console (AGACC) and analyzed by using Expression Console Software (Affymetrix, CA, USA). Data were deposited in the Gene Expression Omnibus database (http://www.ncbi.nlm.nih.gov/geo/query/acc.cgi?acc=GSE118916, GSE118916).

### Data preprocess and differentially expressed genes identification

Probes were converted to gene symbols according to the platform annotation information of the raw data. The expression value for a gene, which was mapped by multiple probes, was acquired by selecting the max value among those probes. Those invalided probes without any gene information were removed. The original CEL data was then started background correction, normalization, and expression calculation by using the R package “affy”. The R package limma (http://www.R-project.org) was used to conduct data normality by log2 transformation. We applied the R package “limma” to identify the differentially expressed genes (DEGs) following the following criteria: (I) |logFC| > 2; (II) P-value < 0.05 and (III) false discovery rate (FDR) < 0.05.

### Gene Ontology and Kyoto Encyclopedia of Genes and Genomes pathway enrichment analysis

Gene Ontology (GO) enrichment analysis and Kyoto Encyclopedia of Genes and Genomes (KEGG) pathway analysis were conducted by using clusterProfiler^6^ to reveal the unique biological significance and key pathways associated with GAC of the DEGs (criteria: p-value < 0.05, significantly enriched). Fisher’s exact test^7^ was used to identify the significant GO terms and pathways and corrected P-value was obtained by Benjamini and Hochberg (BH) false discovery rate (FDR) algorithm. Cytoscape^8^, Enrichment Map^9^, and Gephi^10^ were used for visualization of the network.

### The protein-protein interaction network construction

The Retrieval of Interacting Genes (STRING v10)^11^ (http://string-db.org/) was used to analyze the interactive relationships among DEGs to construct protein-protein interaction (PPI) network and only experimentally validated interactions with a combined score >0.4 were selected as significant. Cytoscape was used to construct the PPI network and Gephi was used to network visualization. The plug-in Molecular Complex Detection (MCODE) was used to select the prime module from the PPI network. The criteria were set as follows: MCODE scores >2 and number of nodes >5. Then the KEGG pathway enrichment analysis of the DEGs from the module was conducted. P < 0.05 was considered to be significant.

### TCGA data acquisition and processing

We searched the GAC cases with both clinical information and gene expression profile from The Cancer Genome Atlas (TCGA) database^12^ by using the R package “OIsurv”. The expression value of each hub genes was defined as either high (expression value >median value) or low (expression value <median value). Survival curves were analyzed by the Kaplan-Meier method^13^, and univariate survival analysis was performed using a log-rank test. Overall survival (OS) was defined as the period between the resection date and death for any cause.

### TCGA data analysis

We downloaded the RNA-seq data and clinical phenotype information of the GAC-related tumor and the adjacent normal tissues from The Cancer Genome Atlas (TCGA) database (https://xenabrowser.net/datapages/). We calculated the expression level of FN1, SERPINE1, and SPARC and conducted the difference significance test between the GAC tumor group and the normal group. We further did the correlation analysis between the hub gene and the disease stage.

### Statistical analysis

The statistical data were processed using SPSS 19.0 software (IBM, SPSS, Chicago, IL, USA). The paired t-test was performed to evaluate the significant difference between tumor tissues and tumor-adjacent normal tissues. The statistical significance of the difference in patients’ overall survival (OS) between the high expression group and low expression group was obtained using the two-sided log-rank test. All p-values were two-sided, and generally, p-values < 0.05 were considered to have statistical significance.

## Results

### Differentially expressed genes in gastric adenocarcinoma

**Figure 1:**
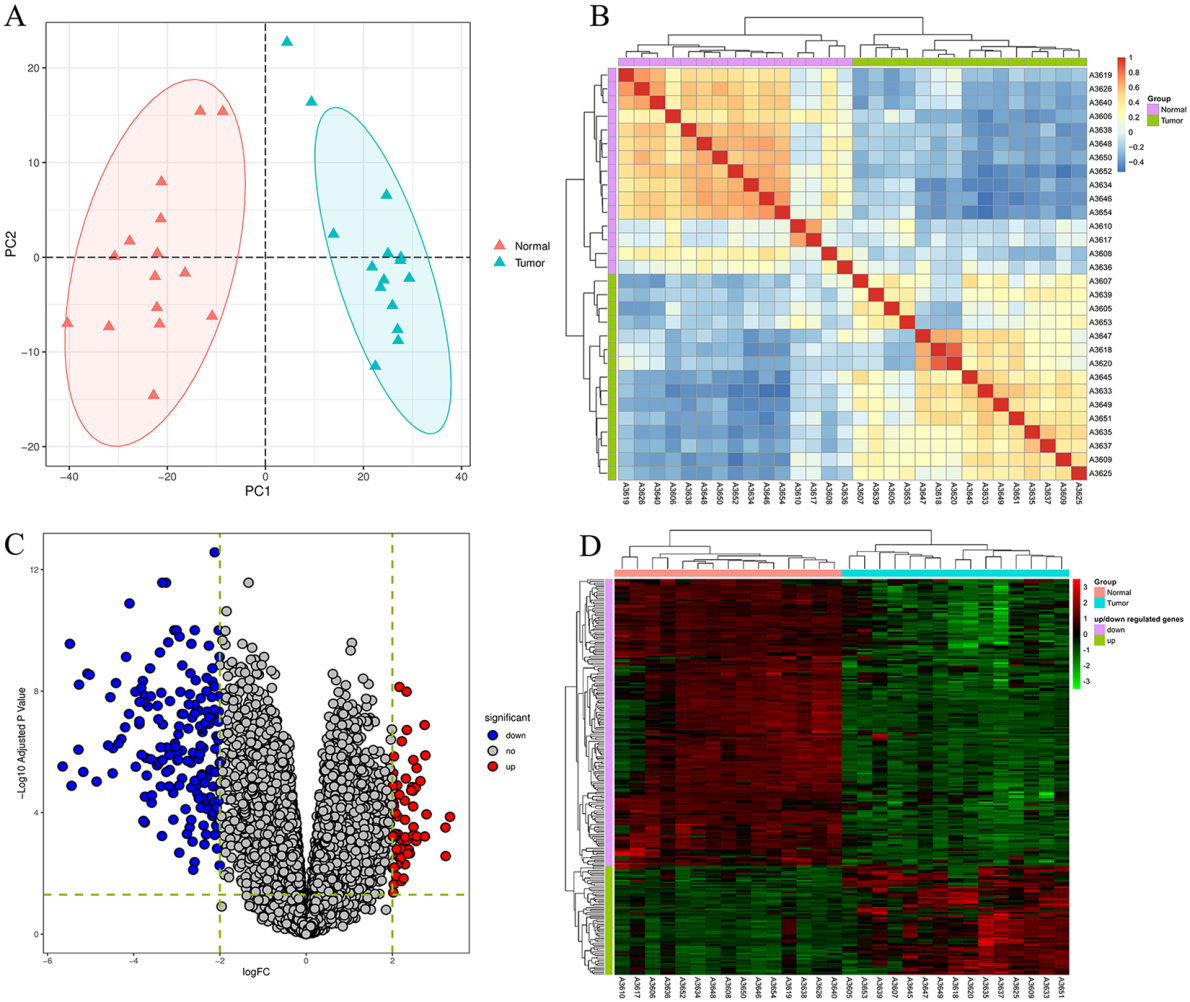
Overview of differentially expressed genes (DEGs) in gastric adenocarcinoma. (**A**) Principal components analysis (PCA) plot for total of 30 samples. (**B**) the pearson correlation matrix among all samples by calculating pairwise correlation coefficient. The redder the color, the higher the correlation coefficient, while the bluer the color, the lower the correlation coefficient. (**C**) Volcano plot of the DEGs. Each circle represents a gene, and red circle represents. (**D**) Heatmap of the 233 DEGs. Each column in the heatmap represents a sample, and each row represents the expression level of a gene. The color scale beside the heatmap represents the raw Z-score ranging from green (low expression) to red (high expression).

Total 15 pairs of GAC tumor and normal samples were analyzed (Fig. 1A) and the basic global gene expression pattern among all samples was denoted by Pearson correlation matrix for calculation of pairwise correlation coefficient (Fig. 1B). Then the first step was to identify the DEGs between these two groups. The classical Bayesian algorithm was applied to identify the DEGs and volcano plots were used for visualizing the DGEs variation between tumor and normal tissues (Fig. 1C). In total, 233 mRNAs displayed differential expression in GAC, including 64 up-regulated mRNAs and 169 down-regulated mRNAs. Each of the top 10 upregulated DGEs and downregulated DGEs were listed in Table 2. Hierarchical clustering analysis showed the expression pattern of DEGs among samples, which suggested that the expression of genes in GAC tumor tissues significantly differ from those in adjacent-normal tissues (Fig. 1D). Detailed information of these up- and down-regulated probes is shown in Supplementary Tables 1 and 2.

**Table 2:** The top10 upregulated DEGs and downregulated DEGs.

prob	Gene_Symbol
**The top10 upregulated DEGs**	
11723174_a_at	FNDC1
11726339_s_at	MAGEA3
11721212_a_at	THBS4
11755955_a_at	FAP
11740290_a_at	HOXC6
11720251_at	CCL18
11715393_a_at	C3
11735643_a_at	RARRES1
11728809_a_at	COL8A1
11742712_a_at	THBS2
**The top10 downregulated DEGs**	
prob	Gene_Symbol
11733660_a_at	CHIA
11728308_at	KRT20
11732742_at	ATP4B
11756545_a_at	GKN2
11723302_a_at	CHGA
11734596_a_at	ATP4A
11744246_at	KCNE2
11715481_a_at	SST
11723940_at	GKN1
11728126_x_at	PHGR1

### Differentially expressed genes enrichment analysis

**Figure 2:**
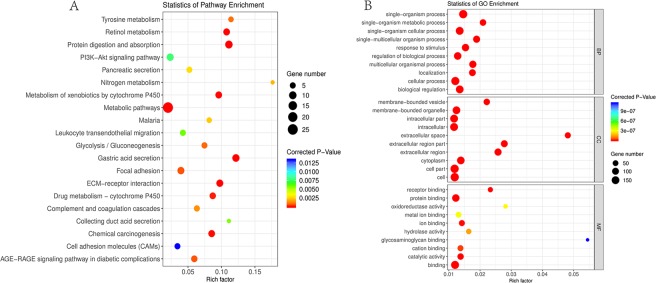
Statistics of functional and pathway enrichment. (**A**) Scatter plot of top 20 enriched KEGG pathways. (**B**) Scatter plot of top 10 enriched GO terms of molecular function (MF), biological process (BP) and cellular component (CC) separately.

**Figure 3:**
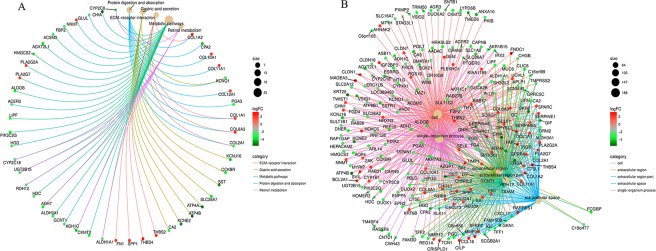
The networks of the top 5 mostly enrichment function or pathways. (**A**) The network of top 5 mostly enriched KEGG pathways. (**B**) The network of top 5 mostly enriched GO terms.

Gene enrichment analysis uses predefined gene sets and ranks of genes to identify significant biological changes or gene co-expression pattern, which could assess the functional associations of target gene set derived from the set of experiments. KEGG analysis results showed that DEGs were mostly enriched in Protein digestion and absorption, Gastric acid secretion, and ECM-receptor interaction (Table 3). Figure 2A shows the TOP20 enriched KEGG pathways. The five top mostly enriched pathways were got to construct a network (Fig. 3A). From the network, the up-regulated genes Human fibronectin gene (FN1), collagen α1 (I) gene (COL1A1) and collagen α2 (I) gene (COL1A2) were supposed to have a direct relationship with ECM-receptor interaction, while the down-regulated gene Somatostatin (SST) was supposed to be associated with Gastric acid secretion. GO analysis results showed that DEGs were mostly enriched in the single-organism process (belonging to a biological process), cellular process (belonging to a biological process), and cell (belonging to cellular component) (Table 4). Figure 2B shows the TOP10 enriched function of molecular function (MF), biological process (BP) and cellular component (CC) respectively. The five top mostly enriched functions were also got to construct a network (Fig. 3B). The detailed information of KEGG and GO enrichment results are listed in Supplementary Tables 3 and 4 separately.

**Table 3:** KEGG pathway enrichment analysis of DEGs associated with GAC.

Gene count	Background number	Rich factor	P-Value	Genes
10	90	11.11%	3.59E-10	COL1A2, CPA2, COL10A1, COL11A1, KCNQ1, COL12A1, PGA3, COL1A1, COL6A3, COL2A1
9	74	12.16%	1.34E-09	KCNJ16, CCKBR, KCNQ1, SST, SLC26A7, ATP4A, ATP4B, KCNE2, CA2
8	82	9.76%	5.48E-08	COL1A2, THBS2, COL2A1, COL1A1, COL6A3, THBS4, SPP1, FN1
25	1243	2.01%	9.98E-08	ALDH1A1, CKMT2, ADH1C, GCNT1, ALDH3A1, ADH7, HDC, RDH12, UGT2B15, CYP2C18, HGD, PIK3C2G, LIPF, ACER2, ALDOB, PLA2G7, PLA2G2A, HMGCS2, AGXT2L1, ACSM3, FBP2, NNMT, GLUL, CHIA, CYP2C8
7	65	10.77%	2.04E-07	ALDH1A1, CYP2C8, UGT2B15, ADH7, ADH1C, CYP2C18, RDH12
7	73	9.59%	4.23E-07	AKR1C1, CYP1B1, AKR7A3, ALDH3A1, ADH7, UGT2B15, ADH1C
7	82	8.54%	8.77E-07	CYP1B1, CYP2C8, ALDH3A1, ADH7, UGT2B15, ADH1C, CYP2C18
6	69	8.70%	4.90E-06	FMO5, CYP2C8, ALDH3A1, ADH7, UGT2B15, ADH1C
8	203	3.94%	3.32E-05	COL1A2, THBS2, COL2A1, COL1A1, COL6A3, THBS4, SPP1, FN1
6	101	5.94%	3.78E-05	COL1A2, SELE, FN1, IL8, COL1A1, SERPINE1

**Table 4:** GO enrichment analysis of DEGs associated with GAC.

GO term	Description	Category	Gene count	Background number	Rich factor	P-Value
GO:0044699	single-organism process	BP	186	12728	1.46%	3.66E-52
GO:0005576	extracellular region	CC	114	4424	2.58%	6.13E-47
GO:0044421	extracellular region part	CC	104	3743	2.78%	7.18E-45
GO:0005615	extracellular space	CC	64	1330	4.81%	3.28E-39
GO:0005623	cell	CC	188	15802	1.19%	1.04E-38
GO:0044464	cell part	CC	187	15776	1.19%	5.27E-38
GO:0009987	cellular process	BP	177	14647	1.21%	4.54E-35
GO:0032501	multicellular organismal process	BP	120	6781	1.77%	1.24E-33
GO:0044763	single-organism cellular process	BP	155	11494	1.35%	2.90E-33
GO:0044707	single-multicellular organism process	BP	110	5822	1.89%	1.50E-32

### PPI network analysis

**Figure 4:**
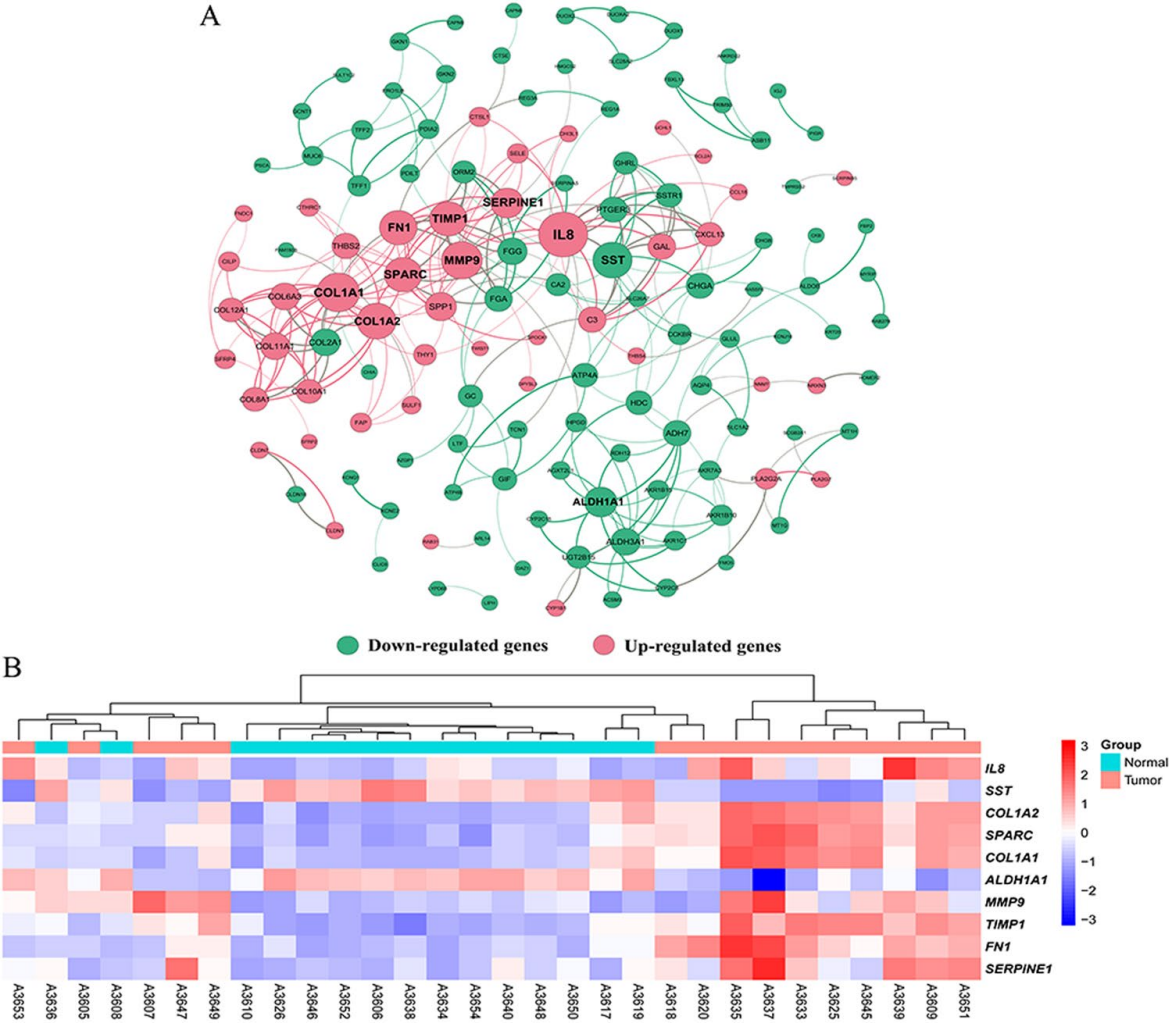
The protein–protein interaction (PPI) network and hub genes. (**A**) PPI network of DEGs. Only experimentally validated interactions with a combined score >0.4 were selected as significant. The nodes were colored according to whether it belongs to up- or down-regulated genes. The thicknesses of those edges were associated with the combined scored. The size of each node is proportional to the number of connections, that is, the degree. (**B**) The expression heatmap of TOP10 hub genes.

**Figure 5:**
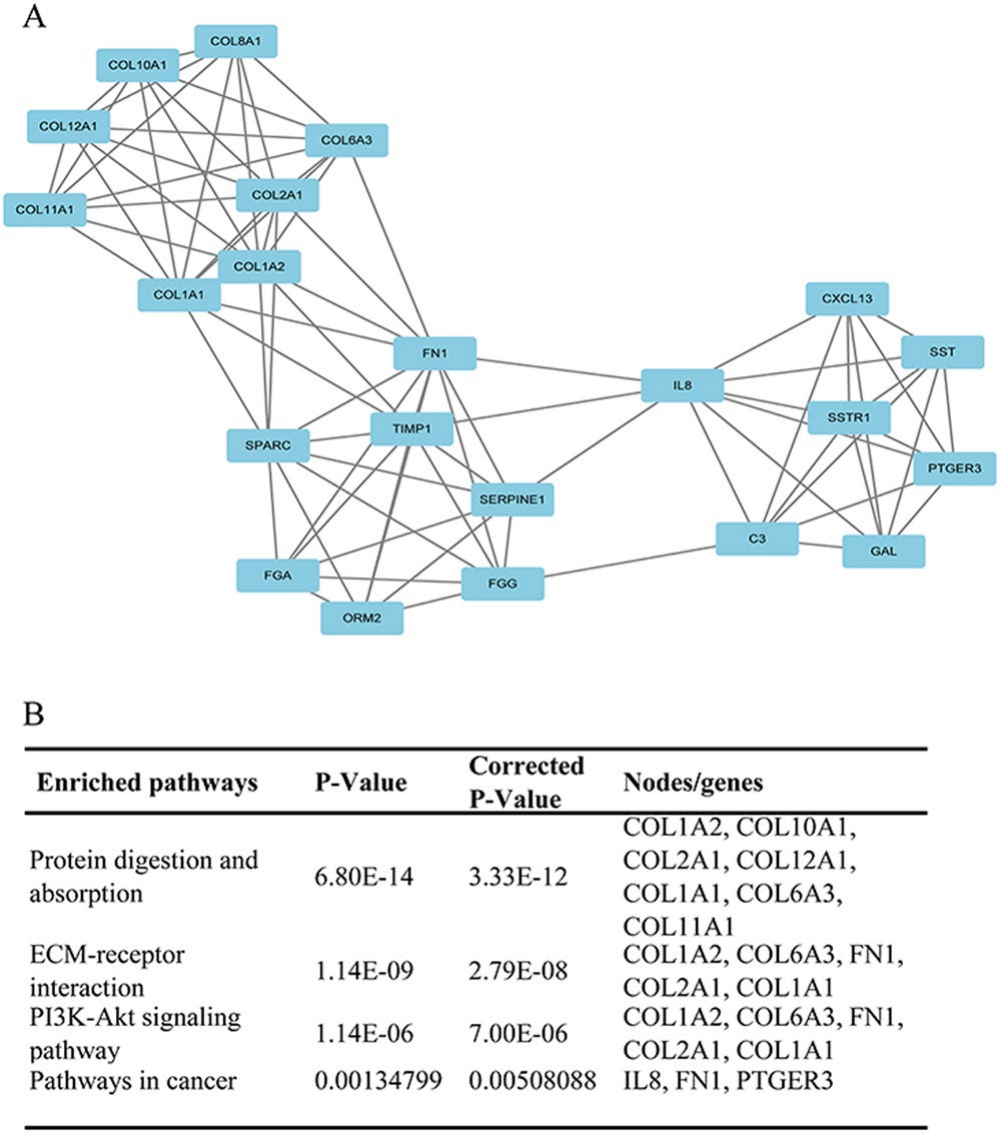
The prime module from the PPI network. (**A**) The sub-network of the main module. (**B**) The enriched pathway of the module.

Based on the STRING database, a co-occurrence network of total 135 nodes and 280 edges was constructed. From the PPI network, the top 10 hub genes with higher degrees were filtered, including Interleukin-8 (*IL8*, up-regulated), Collagen α1 (I) gene (*COL1A1*, up-regulated), Matrix metalloproteinase-9 (*MMP-9*, up-regulated), Somatostatin (*SST*, down-regulated), Collagen α2 (I) gene (*COL1A2*, up-regulated), Metalloproteinases 1 (*TIMP1*, up-regulated), Human fibronectin gene (*FN1*, up-regulated), Secreted protein, acidic, and rich in cysteine (*SPARC*, up-regulated), Aldehyde dehydrogenase 1 (*ALDH1A1*, down-regulated) and Serpin peptidase inhibitor, clade E (*SERPINE1*, up-regulated) (Fig. 4). Among these genes, IL8 had the highest node degree (21). Besides, the prime module was selected (Fig. 5A), and the KEGG pathway enrichment analysis of the genes in the module was analyzed (Fig. 5B). The prime module analysis revealed that the development of GAC was mainly associated with protein digestion and absorption, ECM receptor interaction, the PI3K-Akt signaling pathway and pathway in cancer.

### TCGA dataset analysis

**Figure 6:**
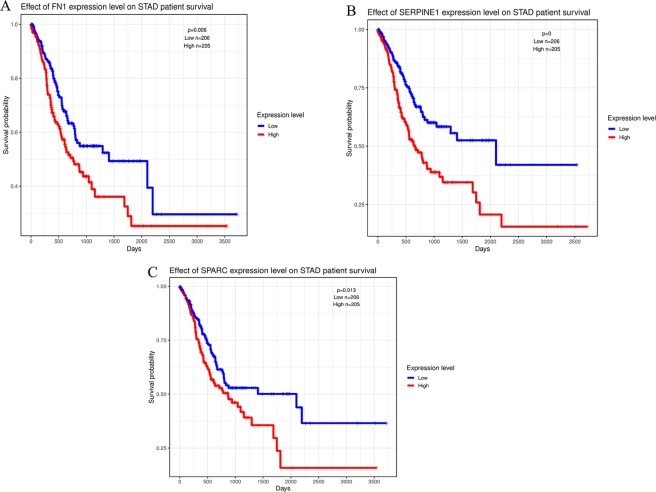
Overall survival analysis in GAC based on the TCGA data as determined by Kaplan-Meier estimates. 411 GAC cases with full data of both clinical and 10 hub gene expression were downloaded from TCGA database. Kaplan-Meier estimates (log-rank test) were made and found the expression of (**A**), FN1; (**B**), SERPINE1 and (**C**), SPARC was significantly affect the prognosis of GAC in overall survival (p < 0.05).

TCGA research analysis could combine extensive genetic studies of human gene expression with the specific disease. GAC cases were divided into high expression (expression value >median value) and low expression (expression value <median value) groups for each key gene. The Kaplan-Meier survival curves of 411 GAC cases showed that FN1, SERPINE1, and SPARC was significantly related to the prognosis of GAC (Fig. 6), while other 7 hub gene was not significantly associated with GAC (Supplementary Fig. 1).

**Figure 7:**
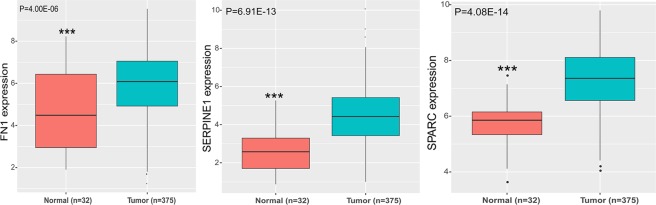
The expression level of FN2, SERPINE1, and SPARC between the normal tissues and tumor tissues. 32 normal tissues and 375 tumor tissues both containing the three gene expression values were collected from the TCGA database.

We further calculated the expression level of FN1, SERPINE1, and SPARC. The results showed that the three hub genes were all extremely significant higher in GAC tumor tissues than the normal tissues (Fig. 7), which were in good consistence with our research. Moreover, we conducted statistical analysis between the expression level of hub genes and disease stage. We found that FN1 (r = 0.21, p = 1.57E-05), SERPINE1 (r = 0.22, p = 5.08E-06), and SPARC (r = 0.14, p = 0.006) all had a significant correlation with CRC disease stage, which indicated that these key genes may have crucial role in GAC progression.

## Discussion

GAC is one of the lethal of malignancies originating in the stomach, which is a kind of heterogeneous disease and consists of various phenotypes and genotypes^5^. Of all, cell cycle plays a key important role in cancer originating including mutation of signaling pathways or mutation of protein-encoding gene, and those relevant cell cycle proteins are thought to have a vital association with cancer progression. Making deeper understanding of the role of cell cycle proteins, including revealing their function mechanisms and finding their relevant upstream or downstream targets, could help us to discover more valuable biomarkers. Fortunately, the development of biotechnology, like microarray, transcriptome sequencing, and proteomics, has provided an advanced way to get the nearly all gene expression information during all stages for any human genomes and lots of potential therapeutic targets have been excavated, including AFP^14^, BCR-ABL^15^, BRCA1/BRCA2^16^, EGFR^17^, HER2^18^ and so on.

In this study, we analyzed the gene expression profiles of 15 GAC tumor tissues and 15 adjacent none-tumor tissues by microarray. We finally identified 64 up-regulated DEGs and 169 down-regulated DEGs. The heatmap of all DEGs showed obviously different between the two groups (Fig. 1D), which demonstrated that each group has its specific gene expression pattern and the samples from the same group seemed to share the similar expression pattern and often participate in a parallel biological process (Fig. 1B). To better understand the interactions among DEGs, we further performed functional enrichment analysis. The GO term enrichment analysis shows that DGEs were mainly involved in cell, extracellular region, extracellular region part, extracellular space, and single organism process (Fig. 3B). As previous studies, the structure of cancer-relevant proteins, like HER2^19^, HER3^20^ has a ligand-activated state in the extracellular region and could interact with other same receptors in the absence of direct ligand binding, which maybe indicate that many DEGs of this study might be transmembrane proteins and may involve in some extracellular signal transduction pathways. KEGG pathway enrichment analysis reveals that DEGs were mainly involved in several cancer-related pathways such as ECM-receptor interaction, protein digestion and absorption, gastric acid secretion and retinol metabolism (Fig. 3A). Gastric acid secretion has been reported to have an association with gastric mucosal healing^21^, which may be involved in the destruction of normal cell proliferation. The previous study pointed out that retinoic acid receptors are frequently having low expression in epithelial gastric cancer^22^.

Subsequently, in order to find the co-existence pattern among all the DEGs, we constructed the PPI network. From the network, 10 hub genes with high degrees were found (Fig. 4). Moreover, we extracted the main module from the PPI network and carried out KEGG pathway enrichment analysis (Fig. 5). The result showed that the family of collagen genes (CLO1A1, COL1A2, COL2A1, etc.) are tight clustered and have directed association with protein digestion and absorption, ECM-receptor interaction and P13K-Akt signaling pathway. Besides the other two pathways, P13K-Akt signaling pathway has been reported to be vital in cell cycle including cell proliferation and gene mutation and is activated in various cancers^23^. The FN1 gene, a glycoprotein involved in cellular adhesion and migration processes, was denoted to have an association with the pathway in cancer.

In order to find the relationship between the target genes and the GAC prognosis, we conducted survival analysis based on the TCGA data, which included 411 GAC cases. The result showed that FN1, SPARC, and SERPINE1 expression were significantly (p < 0.05) associated with GAC prognosis (Fig. 6). The high expression of these three genes often indicates a poor prognosis. The expression level of these three hub genes were all remarkably higher in GAC tumor tissues than the normal tissues (Fig. 7), which indicated these hub genes might have potential impact on the GAC cell progression.

## Conclusion

We identified the hub genes and key pathways associated with GAC by analysis microarray data and provided more information about molecular mechanisms about GAC occurrence and progression, holding promise for acting as biomarkers and potential therapeutic targets. However, further molecular biological experiments including q-RT PCR, colony-formation assay, and flow cytometry analysis are required to confirm the specific functional role and mechanisms underlying the three crucial genes including FN1, SPARC, and SERPINE1 in GAC.

### Human participants and animal rights

All procedures performed in studies involving human participants were in accordance with the ethical standards of the institutional and/or national research committee and with the 1964 Helsinki declaration and its later amendments or comparable ethical standards.

## Supplementary information

Supplementary information

Supplementary Dataset 1

Supplementary Dataset 2

Supplementary Dataset 3

Supplementary Dataset 4
