# Supplementary material for: FN1, SPARC, and SERPINE1 are highly expressed and significantly related to a poor prognosis of gastric adenocarcinoma revealed by microarray and bioinformatics
Source: Sci Rep. 2019 May 24;9:7827. doi: 10.1038/s41598-019-43924-x (PMC6534579; doi:10.1038/s41598-019-43924-x)
Supplement: Supplementary file 1 — Supplementary information [file 41598_2019_43924_MOESM1_ESM.pdf]

FN1, SPARC, and SERPINE1 are highly expressed and significantly related to a poor prognosis of gastric adenocarcinoma revealed by microarray and bioinformatics

Li Li1*, Zuan Zhu1*, Yanchao Zhao1, Qi Zhang1, Xiaoting Wu1, Bei Miao1, Jiang Cao2, Sujuan Fei1

1Department of Gastroenterology, the Affiliated Hospital of Xuzhou Medical University, Xuzhou, Jiangsu 221000
2Department of Hematology, the Affiliated Hospital of Xuzhou Medical University, Xuzhou, Jiangsu 221000
*These authors contributed equally to this work.

Corresponding author: Sujuan Fei, sujuan_fei@126.com. Department of Gastroenterology, the Affiliated Hospital of Xuzhou Medical University, No. 99 West Huaihai Road, Xuzhou, Jiangsu 221002, P.R. China. Tel: 86 516 85805229, Fax: 86 516 85601527.
Or Jiang Cao, jiang_c@126.com. Department of Hematology, the Affiliated Hospital of Xuzhou Medical University, No. 99 West Huaihai Road, Xuzhou, Jiangsu 221002, P.R. China. Tel: 86 516 85802369, Fax: 86 516 85601527.

Supplementary figure:

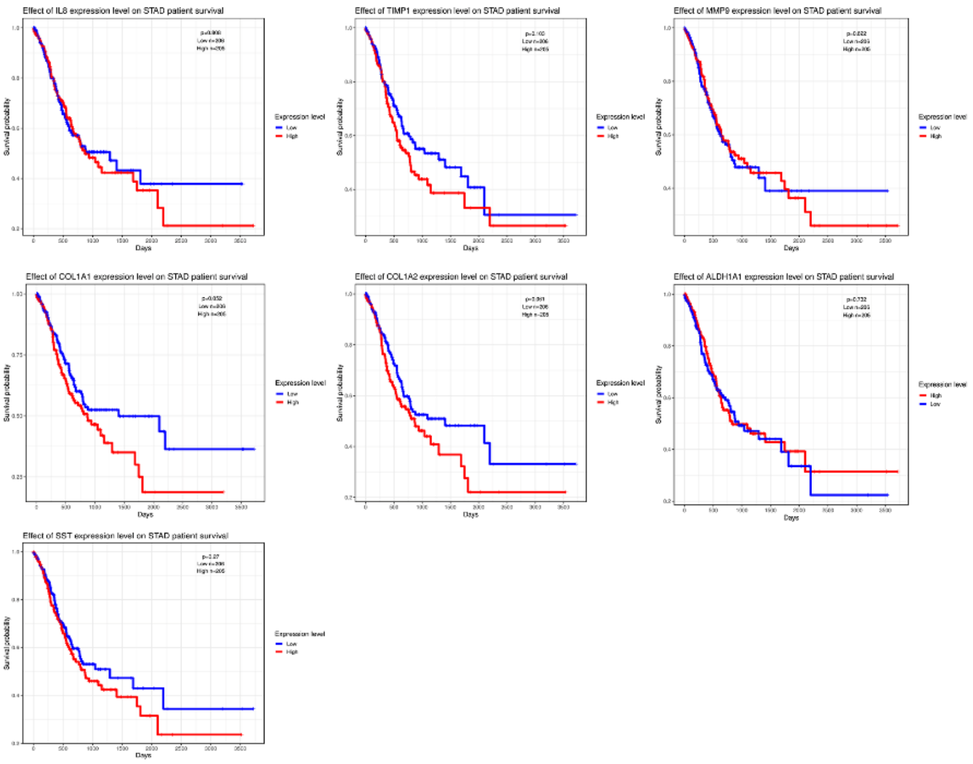

Supplementary figure 1. Overall survival analysis in GAC based on the TCGA data as determined by Kaplan-Meier estimates. 411 GAC cases with full data of both clinical

and 10 hub gene expression were downloaded from TCGA database. The other 7 genes were made by Kaplan-Meier estimates (log-rank test).

Supplementary Table:

Supplementary Table 1. Detailed information of up-regulated probes
Supplementary Table 2. Detailed information of down-regulated probes
Supplementary Table 3. Detailed information of KEGG enrichment results
Supplementary Table 4. Detailed information of GO enrichment results
